# Cross-sectoral video consultations in cancer care: perspectives of cancer patients, oncologists and general practitioners

**DOI:** 10.1007/s00520-020-05467-0

**Published:** 2020-04-21

**Authors:** Theis Bitz Trabjerg, Lars Henrik Jensen, Jens Søndergaard, Jeffrey James Sisler, Dorte Gilså Hansen

**Affiliations:** 1 Research Unit of General Practice, Department of Public Health, University of Southern Denmark, J.B. Winsloews Vej 9A, 5000 Odense C, Denmark; 2 Department of Oncology, Lillebaelt University Hospital, Vejle, Denmark; 3 Danish Colorectal Cancer Center South, Center of Clinical Excellence, Vejle Hospital, Department of Regional Health Research, University of Southern Denmark, Odense, Denmark; 4 Department of Family Medicine, Faculty of Health Sciences, University of Manitoba, Winnipeg, Canada

**Keywords:** User perspectives, Video consultation, Cancer, General practice, Technical fidelity

## Abstract

**Purpose:**

Multidisciplinary video consultations are one method of improving coherence and coordination of care in cancer patients, but knowledge of user perspectives is lacking. Continuity of care is expected to have a significant impact on the quality of cancer care. Enhanced task clarification and shared responsibility between the patient, oncologist and general practitioner through video consultations might provide enhanced continuity in cancer care.

**Method:**

We used descriptive survey data from patients and doctors in the intervention group based on a randomised controlled trial to evaluate the user perspectives and fidelity of the intervention.

**Results:**

Patients expressed that they were able to present their concerns in 95% of the consultations, and believed it was beneficial to have both their doctors present in 84%. The general practitioner and oncologist found that tripartite video consultation would lead to better coordination of care in almost 90% of the consultations. However, the benefits of handling social issues and comorbidity were sparser. Consultations were not accomplished in 11% due to technical problems and sound and video quality were non-satisfactory in 20%.

**Conclusion:**

Overall, multidisciplinary video consultations between cancer patient, general practitioner and oncologist were feasible in daily clinics. Initial barriers to address were technical issues and seamless planning. Patients reported high satisfaction, patient centredness and clarity of roles. General practitioners and oncologists were overall positive regarding role clarification and continuity, although less pronounced than patients.

**Trial registration:**

www.clincialtrials.gov, NCT02716168.

## Introduction

Multidisciplinary video consultations are one method to improve coherence and coordination of care in cancer patients, but knowledge of user perspectives is lacking. Continuity of care is expected to have a significant impact on the quality of cancer care and the patient quality of life [1]. Therefore, interventions addressing gaps in continuity may improve patients’ satisfaction, health outcomes [2] and lower health care needs [3]. In line with guidelines, coordination of care, collaboration across health care sectors and involvement of general practitioners (GPs) are regarded as essential for continuity of care [4]. However, continuity of cancer care still poses a substantial challenge to accomplish [5], and general practitioners (GPs) are often disconnected from care planning [6].

Consequently, patient [7] and oncologist [8] can be uncertain about the GP’s competence and role. To provide effective cancer care in the future, new models supporting the exchange of knowledge and task clarification between oncologists and GPs are needed, continuously involving the patients’ needs [9]. Bringing them together in a shared consultation might be a powerful solution.

Due to geographical reasons and shortage of time, shared consultations are not feasible as part of routine cancer care. Video consultations have become increasingly common [10, 11], and video-based communication may be an alternative solution to connect health professionals sitting apart [12–14]. Seeing each other is essential for building and establishing professional relationships and can be accomplished through video [15]. We are not aware of studies exploring video consultations bringing a patient together with his/her GP and oncologist. However, for years, video solutions have been used for multidisciplinary team meetings in cancer treatment planning [16]. Recent trials have included GPs [17] or patients [18, 19], but not simultaneously. Moreover, video solutions have been used in palliative care, and studies have highlighted how video consultations can contribute to effective and inclusive communication, and facilitate a feeling of security, trust and relationship building although sitting apart [20, 21]. We therefore developed the Partnership Study aiming to test multidisciplinary video consultations between a cancer patient, oncologist and GP in a randomised design [22]. In this paper, we aim to analyse video consultations from the user’s perspective (patients and doctors), based on three surveys of patients enrolled in the intervention group, and their oncologists and GPs. We evaluated key elements of the intervention: continuity, patient involvement and sharing of knowledge between health professionals. We also evaluated technical quality by surveying patients and oncologists.

## Method

This study is based on survey data on user perspectives from cancer patients, their oncologists and GPs who participated in video consultations in the Partnership Study: a randomised controlled trial (RCT) evaluating the Partnership Intervention. The RCT has been described in more detail elsewhere [22]. Descriptive data covering patient age and gender, cancer localisation and intention of treatment were retrieved from hospital-based electronic patient records. Socio-economic characteristics (education, employment status, living with a spouse and children at home) were included in the patient survey. Descriptive data regarding oncologists and GPs were retrieved from the corresponding surveys.

### The Partnership Intervention

Patients in the intervention group received “the Partnership Intervention” in addition to “usual care”. The oncologist invited the GP to take part in one of the patient’s consultations during the ongoing oncological treatment. They were brought together using internet video, allowing them to see and hear each other although sitting apart. The patient could choose to be with the oncologist or the GP. The consultation was planned as early as possible within 12 weeks from time of inclusion, corresponding to a maximum of 15–18 weeks after the first appointment at the Department of Oncology. The consultation was conducted as part of the planned standard programme at the hospital, but if the patient chose to be located at the GP’s office, further consultation was scheduled. Consultations were booked 3–6 weeks in advance within regular clinic hours.

**Fig. 1:**
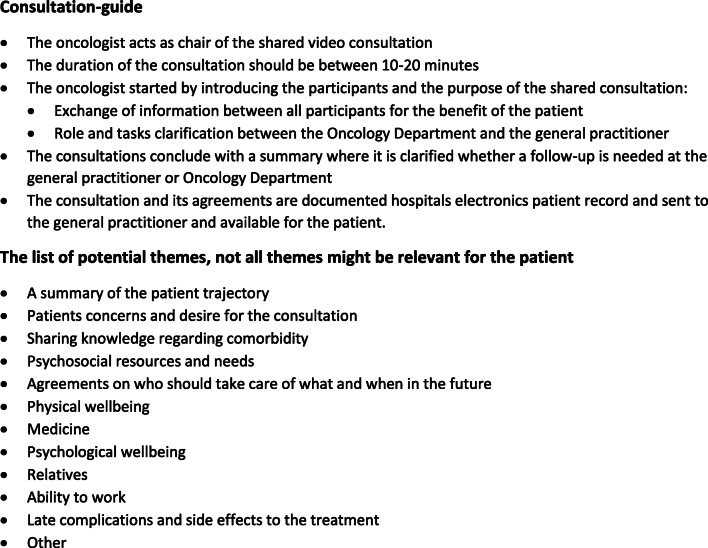
The consultation guide to GPs and oncologists, including themes potentially relevant for the consultation

Before each consultation, oncologists and GPs received specific information about the aim of the consultation, including a “consultation-guide” with themes that may be relevant for their dialogue (Fig. 1). It was emphasised to the doctors that not all themes might be relevant for all patients. The three experts in the consultation, the GP, oncologist and a patient, should bring up the most important issues according to their knowledge. The guide was sent by email to the GPs as part of study information and presented to the oncologist before the consultation. The consultation guide was inspired by the Calgary-Cambridge Guide, supporting doctor-patient communication training [23], a literature search focused on unmet needs for cancer patients [24] and user perspectives of participating in video consultations in health care [12, 25, 26]. The development included feedback from a user panel of GPs and oncologists along with “The Patient and Relatives Council” at the hospital.

In line with the consultation guideline, the oncologist chaired the consultation, wrote a summary to be sent electronically to the GP and included in the hospital’s electronic patient record. The summary was available for the patients at Sundhed.dk, an online portal where patients can read their entire medical record from secondary care.

### Setting and participants

The Partnership Study was performed in cooperation between the Department of Oncology, Lillebaelt University Hospital, Denmark and general practices in the Region of Southern Denmark. Annually, around 1300 cancer patients are referred for chemotherapy at the department. The hospital’s catchment area includes about 750,000 citizens, though depending on the different cancer diagnoses. Five hundred GPs work in approximately 300 general practice medical centres comprising 1–8 physicians in the region. In Denmark, all citizens are eligible for free medical service at public hospitals and in primary care. GPs are located in their own local offices close to where patients live [27]. GPs are gatekeepers to more specialised health care services, and more than 98% of the population are registered with a specific general practice [27].

All participating GPs were family medicine specialists with private practices. They had an average length of service of 15 years (range 3–34 years), and 52% were females. The oncologists included 13 oncology medical specialists and one doctor in training for oncology (57% female).

Cancer patients were eligible regardless of their cancer diagnosis. We invited all 18+-year-old newly diagnosed patients if receiving oncological treatment for the first time at the department. Participants should have an expected survival time of more than 7 months as assessed by an oncologist and be able to speak and read Danish. Eligible patients were handed study information in connection with their second oncological treatment session.

When a patient was allocated to the intervention group, a research nurse at The Research Unit for General Practice, Odense contacted the GP to invite him/her to take part in the study. If the GP did not have the video equipment required, the research nurse arranged free installation by a technician from the Health Innovation Centre of Southern Denmark. When ready, the GP’s secretary was contacted by an oncology nurse coordinator to schedule the video consultation within 3–6 weeks.

**Fig. 2:**
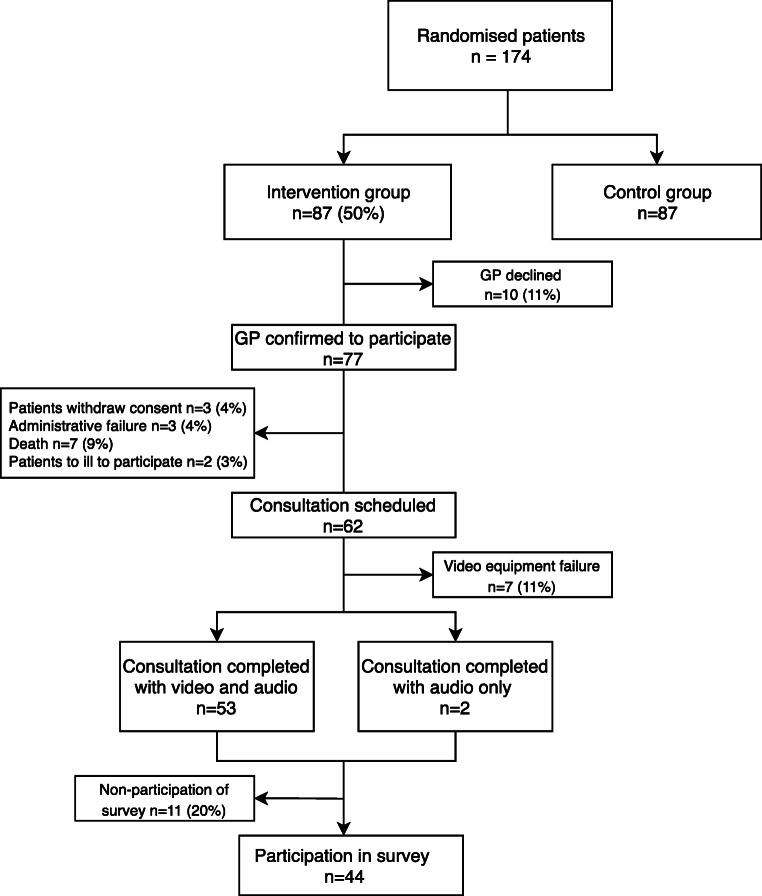
Flowchart of participants enrolled in the randomised controlled trial “The Partnership Project”. From randomisation to survey participation

The sample for this paper on process evaluation was drawn from the ongoing RCT, and consists of 87 patients allocated to the intervention group between June 2016 and April 2019 [22]. At that time, all previous patients in the allocation sequences had completed the intervention, or a note was made in the record why the intervention would never be completed. Of the 87 patients allocated to the intervention group, 55 completed the intervention (Fig. 2).

### Technical issues

The consultations were accomplished using a virtual meeting room. The picture-in-picture feature showed the speaking participants in full screen.

At the hospital department, we used the Cisco® TANDBERG™ E20 screen system. The GPs used the Cisco® Jabber system with an external combined microphone/speaker and a small webcam attached on top of their computer screen.

All data were processed through the Secure Servers. To avoid technical problems and misunderstandings, project manager TBT contacted the GP office 1 day in advance halfway through the inclusion period, to repeat how a virtual meeting room works and the number to be dialled. To further smooth the process and rate of success, TBT was present “in” the virtual meeting room until video contact was established.

### Outcome measures

Patients, GPs and oncologists had separate questionnaires (Tables 2, 3 and4).

Immediately after the consultation, the oncologists evaluated their experiences. A project nurse entered their responses in RedCap® [28], which prompted the issuing of surveys to patients and GPs. Within 24 h, they were each emailed a short letter including link and password, giving them access to their electronic survey. Two reminders were sent by email and a third and last in paper format with a return envelope to improve response rates. Furthermore, patients and GPs were given the option of receiving the survey in paper format.

In the absence of validated survey instruments covering the experience of patients and professionals from tripartite consultations, we prepared ad hoc questions evaluating key elements of the intervention: continuity, role clarification, patient involvement and sharing of knowledge between professionals. Patients and oncologists were also asked to evaluate the technical aspects of the video consultation.

The themes and items were based on the literature [24, 29, 30] and piloting of the intervention [22]. Items covering the technical aspects of video consultation were mainly inspired by the Australian College of Rural & Remote Medicine [25].

Patients were asked about four themes in addition to technical evaluation (Table 2): patient involvement, role and responsibility, satisfaction and the setting of the consultation. In addition to a technical evaluation of quality of picture and audio, consultation duration and patient location, the oncologists evaluated five themes (Table 3): continuity, sharing of knowledge, roles and responsibility, possible relief to the department and overall satisfaction. The GPs evaluated four themes (Table 4): continuity, sharing of knowledge, roles and responsibility and the exchange of information between sectors.

The oncologists and GPs answered using a four-point Likert scale from “very much” to “not at all”. Oncologist evaluation regarding technical solution was answered on a three-point Likert scale from “poor” to “good”. Patient questionnaires, including technical evaluation, were answered in a different four-point Likert scale from “agree” to “disagree” with the option of “do not know”. These scales correspond to what has been used in other studies in a Danish cancer setting to measure cross-sectoral cooperation [31] and technical aspects of video consultations [25].

### Statistical analysis

We conducted a descriptive analysis, including response distribution (numbers and percentages) and measures of central tendency. Answers were dichotomised. Two categories were considered in favour of the question: for patients, “agree” and “partly agree” and for doctors “very much” and “partly”. We also calculated dispersion, including 95% confidence intervals (CI) for consultation times. For comparison in time between patient locations, we applied a Wilcoxon rank-sum test as data were not normally distributed.

## Results

From June 2016 until 15th of April 2019, 174 patients were enrolled in the Partnership Study, including 87 (50%) randomly allocated to the intervention group (Fig. 2). From the intervention group, 62 patients could potentially complete the consultation. However, in seven of these cases (11%), video equipment failures caused cancellation. Therefore, this study is based on results from 55 joint consultations. Scheduling proved to be time-consuming for the oncology coordinators, and extra resources were allocated to ensure seamless planning. Baseline characteristics of the patients allocated to the intervention group are presented in Table 1. In the majority of cases (*n* = 47; 85%), the patients were located at the hospital, whereas eight patients (15%) were at the GP’s office. The 55 video consultations were completed by 52 GPs and 14 oncologists. Three GPs participated in two consultations, and the remainder in one. The mean number of consultations per oncologist was four (range 1–9).

**Table 1:** Baseline characteristics of patients allocated to the intervention group of the Partnership Intervention, and the subgroups who completed the intervention, and answered the survey subsequently

Patient characteristics	Allocated to intervention (*n* = 87) N (%)	Completed the intervention (*n* = 55) N (%)	Completed the survey (*n* = 44) N (%)
Mean age, years (SD)	68 (9.5)	66 (9.8)	65 (10,2)
Gender (males)	44 (51)	27 (49)	21 (48)
Education			
Primary school	48 (56)	30 (55)	22 (50)
High school	10 (12)	7 (13)	6 (14)
Higher education 3–4 years	18 (21)	14 (25)	13 (30)
Higher education 5 years	10 (10)	4 (7)	3 (7)
Living with spouse	64 (74)	45 (82)	37 (84)
Children at home	11 (13)	8 (15)	8 (18)
Employment status			
Retirement	48 (56)	32 (58)	23 (52)
Working	28 (33)	20 (36)	18 (41)
Other	10 (12)	3 (5)	3 (7)
Primary cancer			
Breast	9 (10)	9 (16)	6 (14)
Lung	32 (37)	22 (40)	17 (39)
Colorectal	34 (39)	15 (27)	15 (34)
Other Prostate Pancreatic Gynaecological Cholangiocarcinoma	12 (14)	9 (16)	6 (14)
Intention of treatment			
Potentially curative	53 (61)	33 (60)	29 (66)
Non-curative	34 (39)	22 (40)	15 (34)
Comorbidity (reported by patients)	37 (44)	22 (40)	19 (43)

The mean duration of all consultations was 15 min (CI 14–16). There was no significant difference in the duration of the consultation when the patient was present at the GP’s office or the Department of Oncology (15 (11.9–18) vs. 15 (13.9–16.1) min, *p* = 0.3).

### Evaluation by patients

The response rate of the patient survey reached 80%. Table 2 shows the responses in detail. Based on the dichotomisation of the responses, 95% of the patients were allowed to present their needs in the consultations. They became more aware of the role of the oncologists and GPs in the trajectory in 91% and 86% of consultations respectively.

**Table 2:** Patient evaluation of a video-based consultation, including GP and oncologist (*n* = 44). The table shows the four themes: (1) patient involvement, (2) role and responsibility. (3) satisfaction and (4) the setting of the consultation

	Agree N (%)	Partly agree N (%)	Partly disagree N (%)	Disagree N (%)	Do not know N (%)
Patient involvement					
I was allowed to present the issues that worried me the most.	42 (95)	0	1 (2)	0 (0)	1 (2)
Role and responsibility					
I have become more aware of the role of the Department of Oncology, Vejle Hospital in the treatment.	34 (77)	6 (14)	1 (2)	1(2)	2 (5)
I have become more aware of my GP’s role in the trajectory.	27 (61)	11 (25)	2 (5)	2 (5)	2 (5)
I feel more confident about whom to contact.	33 (75)	8 (18)	1 (2)	1 (2)	1 (2)
Satisfaction					
It was useful for me to have the trajectory summed up.	35 (80)	3 (7)	3 (7)	1 (2)	2 (5)
It was helpful for me to have the planned treatment explained.	34 (77)	7 (16)	1 (2)	1 (2)	1 (2)
I believe that it was useful for my GP to have the planned treatment explained.	36 (82)	5 (11)	1 (2)	1 (2)	1 (2)
It was helpful to have a consultation in which both my GP and oncologist participated.	37 (84)	5 (11)	0	2 (5)	0
I believe a video conversation can be useful to me again at a later date.	31 (70)	9 (20)	2 (5)	2 (5)	0
The setting of the consultation					
I understood the role of each participant in the video consultation.	38 (86)	4 (9)	0	2 (5)	0
I felt comfortable during the video consultation.	41 (93)	2 (5)	0	1 (2)	0
The purpose of the video consultation was clear.	31 (70)	10 (23)	1 (2)	2 (5)	0

Ninety-three percent of the patients also became more confident in whom to contact with a given problem. Regarding “satisfaction”, 95% believed it was helpful to have a consultation with both oncologist and GP, and 90% would like to have a similar video consultation later in their trajectory. The “setting” theme showed that close to all patients felt comfortable during the consultation (98%), understood the role of each participant within the consultation (95%) and found the purpose of the consultation clear to them (93%).

### Evaluation by oncologists

Based on a response rate of 100%, the results include 15 oncologists’ evaluations of 55 consultations. Table 3 shows the responses in detail. Based on the dichotomised responses, a total of 86% of the consultations were found to contribute to a more coherent trajectory and deemed useful in 76% of the cases. The oncologists retrieved valuable knowledge about the GPs’ role in the patient trajectory from two out of three consultations, and in 37% valuable knowledge from the GP regarding comorbidity. Regarding “roles and responsibility”, actual agreements between the doctors were made in 80% of the consultations. In 40% of the cases, the oncologists believed the consultation could yield relief for the department.

**Table 3:** The oncologists’ evaluation of video consultations (*n* = 55). The table shows the five themes: (1) continuity, (2) sharing of knowledge, (3) roles and responsibility, (4) relief to the department and (5) overall satisfaction

	Very much N (%)	Partly N (%)	Slightly N (%)	Not at all N (%)
Continuity				
The video consultation can help create a better and more coherent course for the patient.	18 (33)	29 (53)	8 (15)	0
Sharing of knowledge				
I gained knowledge about his/her role in the trajectory.	16 (29)	20 (36)	13 (24)	4 (7)
I gained knowledge about comorbidity.	8 (15)	12 (22)	15 (27)	20 (36)
I gained knowledge about psychological problems.	2 (4)	22 (40)	12 (22)	19 (35)
I gained knowledge about social problems.	4 (7)	17 (31)	11 (20)	21 (38)
Roles and responsibility				
The consultation helped to focus on topics that are often overlooked.	9 (16)	13 (24)	23 (42)	10 (18)
The consultation resulted in specific agreements on roles and responsibilities.	21 (38)	23 (42)	11 (20)	0
Relief to the department				
The agreements will be able to yield relief for the department.	7 (13)	15 (27)	27 (49)	6 (11)
Overall satisfaction				
All in all, it was a useful consultation.	20 (36)	22 (40)	13 (24)	0

### Evaluation by general practitioners

We reached a response rate for GP surveys of 71%. Table 4 shows the responses in detail. Based on the dichotomisation of the responses, a total of 90% of the GPs found that the consultation could give a more coherent course for the patient. In 69% of the cases, the consultation helped to clarify their role during the trajectory and in an equal number enabled presentation of helpful information from the hospital that was not previously present. The GPs found that the consultation helped them to handle physical consequences and side effects of chemotherapy in 61 and 54% of cases respectively, and lead to better treatment of comorbidity (41%) or would help them in to take care of psychological (36%) and social issues (33%) in a lower number of cases.

**Table 4:** Evaluation by general practitioners of the video consultations including *n* = 39. The table shows the four themes: (1) continuity, (2) sharing of knowledge, (3) roles and responsibility and (4) exchange of information between sectors

	Very much N (%)	Partly N (%)	Slightly N (%)	Not at all N (%)
Continuity				
The video consultation can help create a better and more coherent course for the patient.	19(49)	16(41)	4(10)	0
Sharing of knowledge				
The video consultation helped me better handle side effects to chemotherapy.	8(21)	13(33)	11(28)	7(18)
The video consultation helped me better handle the physical consequences of chemotherapy.	6(15)	18(46)	12(31)	3(8)
The video consultation helped me better handle psychological problems.	5(13)	9(23)	18(46)	7(18)
The video consultation helped me better handle social issues.	5(13)	8(21)	17(44)	9(23)
The video consultation helped me better handle comorbidity.	3(8)	13(33)	12(31)	11(28)
Roles and responsibility				
The video consultation helped clarify my role in the patient’s ongoing treatment.	13(33)	14(36)	11(28)	1(3)
Exchange of information between sectors				
Before the video consultation, I had received information from discharge summaries that met my needs.	20(51)	16(41)	3(8)	0
The video consultation gave me useful information that complements previous discharge summaries from the department.	13(33)	14(36)	10(26)	2(5)

### Technical evaluation

Of the 53 consultations completed as intended in the protocol, the oncologists assessed the sound quality and video quality as good in 80% and 76% of consultations respectively.

Patients were satisfied with the technical aspects, “hearing” and “seeing” participants clearly in 93% and 95% of the consultations respectively. Furthermore, 98% of patients found that enough time was set aside. The number of cancellations due to technical failure was 7 out of 62 (11%), and two were completed using a telephone (Fig. 2). In 20% of consultations, the participants experienced a non-satisfactory sound and video quality.

## Discussion

This study showed that when it is possible to bring a cancer patient, GP and oncologist together for a multidisciplinary, video-based consultation, a very high degree of user satisfaction is reached from all three participants. During these tripartite consultations, patients believed they were able to be involved by presenting their needs and concerns, roles and tasks became more apparent to both patients and professionals, and knowledge was shared between health sectors. All perceived a contribution to better continuity of care and thus better health care [1, 2, 32].

Patients experience continuity of care by having confidence in the care path and trust in the providers [33]. However, cancer patients often serve as their own care coordinators and navigate the many steps in their trajectory [34]. In the current study, almost all patients became more confident in the different roles the health professionals play and more confident in whom to contact with a given problem, thereby laying the foundation for confidence in navigating in their pathway and establishing a more coherent trajectory.

The perspectives of GPs on roles and task clarification were deemed essential for enhanced continuity in a review by Lawrence et al. [35]. According to the authors, there is a need for more correspondence between sectors, which could be achieved by electronic summaries, but preferably by personal interaction through meetings between doctors involved in care provision. The current study shows that roles and responsibility between sectors could be accomplished through joint consultations.

When care for cancer patients occurs in different settings, incomplete sharing of information between primary and speciality care providers has been described, and cancer patients described this experience as like “falling through the cracks” [36]. Comorbidities have often been mentioned as problematic and incompletely handled during cancer treatment, since expectations and agreements are seldom reached [37]. In contrast to this literature, bringing together patient, GP and oncologist resulted in a highly satisfactory transfer of knowledge between professionals regarding comorbidity. In line with a previous survey among Danish cancer patients [38], 43% of the participating patients reported having comorbidity. Information about comorbidity was deemed satisfactory in 41% and 37% of the consultations as perceived by the GP and oncologist respectively. Therefore, our results suggest that when comorbidity was present, the consultation improved sharing of knowledge and information between providers.

Video-based consultations require easy-to-use, high quality, reliable, safe and legal communication equipment [12, 14]. In line with a review by Kitamura et al. [13], we found that when the establishment of a digital connection succeeded, both oncologists and patients were satisfied with the quality of the audio and video. The study also shows that even with the participation of two medical specialists, the patient felt comfortable and the purpose of the consultations was clear to them.

In line with studies from a Cochran review [39], technical failures appeared in 11% of planned consultations. Failures could influence the fidelity and effect of the intervention and may act as a barrier to future implementation.

### Strength and limitations

Our response rates from patients, GPs and oncologists of 80%, 71% and 100%, respectively, are considered highly satisfactory and reduced the risk of selection bias [40].

The lack of validated scales is an explicit limitation of the study compared with instruments with established measurement properties [41]. However, we relied on well-known Likert scales and inspiration from questionnaires previously used in a Danish cancer setting [31, 38] and tested the questionnaires in our piloting [22].

Concerning the generalisability of study results, it is important to acknowledge the strategic work that has already been done at the trial hospital. The hospital has been known for years as being innovative regarding cross-sectoral cooperation [42], shared decision-making [43] and patient-centred communication [44]. Therefore, the oncologist may be more open-minded towards sharing knowledge and decisions with GPs, and more focused on communication and patient involvement than oncologists in general. Likewise, the GP may have been more in touch with their workload and the potential of cooperation.

Social desirability bias may have resulted in over-optimistic responses from participants with personal interests in portraying the intervention positively; LHJ as a project manager and the oncologists as his colleagues; patients being thankful to the oncologist for an invitation and to the two health care professionals for taking their time. Furthermore, it could be argued that our information was biased when only relying on the successful cases.

### Perspectives

Despite fast-growing technology within video communication [10, 11], there is still a gap in understanding and troubleshooting when the systems do not work. Taking into consideration the stress and discontent that can be caused when system failures occurred, our results underline the need for easy-to-understand instructions and a hotline as technical issues is common.

Scheduling consultations, including participants from different settings, is logistically challenging but manageable for non-acute problems [45]. In our study, all consultations were embedded in regular clinic hours. For oncology, consultations are usually 30 min and general practice 15-min slots. The video consultations averaged 15 min, thereby integrating them in the everyday clinic at both specialties and allowing the oncologists enough time for clarifying oncology specific topics after the video consultation. To enhance the success of the video consultations, dedicated staff was closely linked to the coordination and technical fidelity. A project member was engaged at the hospital as well as general practice level. In the current study, coordination and technical support was quite comprehensive. As technology gets mere embedded in clinical practice [45], we suggest that in the near future, easy-to-use video technology is fully integrated into both settings, and coordination tasks are reduced to a low level. However, increasing the quality of cancer care may cost. A health economic evaluation may contribute to the question “was it worth the time?”

Results from the randomised design are an important next step before implementation. Surveys on oncology nurses and relatives, as well as focus group interviews with the different participants, may broaden our understanding of the concept. Furthermore, interviews with GPs and patients focusing on reasons for non-participation may be relevant for future research.

## Conclusion

This novel approach of cross-sectoral communication with cancer patients has shown that bringing a cancer patient, GP and oncologist together for a video-based consultation was feasible in clinics, although initial barriers, such as technical issues and seamless planning, need to be addressed. Consultations contributed to enhanced continuity of care as perceived by the users. Moreover, doctors experienced confidence in the roles and responsibility for their care.
